# Transactions of the Gynæcological Society of Chicago

**Published:** 1888-12

**Authors:** 


### TRANSACTIONS OF THE GYNÆCOLOGICAL SOCIETY OF CHICAGO.

The regular meeting was held September 28, 1888, the President, Henry T. Byford, M. D., in the chair.

### RECENT MODIFICATIONS OF BOZEMAN'S UTERINE CATHETER.

Professor W. W. JAGGARD said that it was difficult to clean and sterilize many of the double-current tubes commonly used for the purpose of intra-uterine irrigation. He begged to call attention to three models of Bozeman's uterine catheter as variously altered, in important particulars, respectively by Breisky, Breuss, and Piskacêk. Bruess' instrument resembled the modification by Chrobak and Fritsch, but permitted the use of a much smaller uterine tube—four millimetres in diameter—of obvious advantage in the irrigation of the non-gravid uterus after curettement. Although the uterine tube can be removed from the conduit-pipe, this instrument requires care in cleaning. Piskacek's model is an almost perfect instrument. It has been in the shops for some time, and has answered the purposes for which it was designed. This catheter is identical in principle, and nearly so in detail, with the "Asceptic Two-way Uterine Catheter" exhibited by Dr. H. A. Kelly to the Obstetrical Society of Philadelphia, March 1, 1888. A cut of the instrument shown by Dr. Kelly, appearing in the July number of the *American Journal of Obstetrics*, etc., may serve to illustrate Piskacêk's device.

### SELF-RETAINING DRAINAGE-TUBES FOR PELVIC ABSCESSES OPENING INTO THE RECTUM.

The PRESIDENT said: I wish to exhibit some self-retaining drainage-tubes that I devised for draining pelvic abscesses discharging into the rectum. One of these was successfully used in the cure of an abscess that I opened from the rectum. I used another successfully in a double uterus with a single cervix, in which the retained blood on one side had to be excavated a little in front of and to the right of the os. It was used until the pocket formed by the previous distention (hematometra), and that extended below its outlet at the internal os, had become obliterated by slow contraction. I am now using one to drain the urine from an inflamed bladder. They are curved so that the concave side represents in profile a semicircle, the convex side an arc of about ninety degrees. They are made of silver, or of nikel-plated metal.

PEDICLE-FORCEPS FOR VAGINAL OÖPHORECTOMY.

I have here also a pedicle-forceps, to be used in vaginal oöphorectomy. I always use ligatures when practicable, but in an operation for a small adherent dermoid, at which I assisted Dr. Newman, neither of us could apply a ligature satisfactorily. I lent him an instrument similar to this perfected one, that he left on the pedicle for two days with good results. Both ends are bent in opposite directions.

The perfected forceps has been since used in a case of vaginal ovariotomy with good results.

MODIFICATION OF SIMS' NEEDLE-FORCEPS.

Here is a new needle-forceps, or rather a modification of the Sims' pattern. They are made so as to grasp full curved needles as well as straight ones; they have an extension on the end to enable the operator to use the force of the whole hand, while the thumb and two first fingers guide the motions of the forceps just as with Sims' instrument.

PAPILLOMA OF THE BROAD LIGAMENT INFILTRATED WITH TUBERCULOUS FOCI.

Dr. McArthur will exhibit, through the microscope, a very interesting specimen of papilloma that I removed six weeks ago from a girl twenty-one years of age. She has had abscesses in different parts of the body since she was three or four years old, up to two years ago. Her front teeth were decayed, and her general appearance was cachectic. She also had a complete procidentia of the uterus. In spite of the fact that she had been kept in bed nearly a month, there was still a temperature of 101° and 102°, even going as high as 103° in the afternoon, not due to the prolapse, as the uterus had been replaced and did not come out while she kept her bed. She did not tolerate a thorough examination, so I put her under either and found a small tumor about the size of a goose egg on the posterior surface of the broad ligament. I took it to be an abscess of the ovary, although not clear about it. Her pulse was very weak. At the operation I found this mass completely invested in dark gray, adherent omentum. I could not get it out without ligating and cutting away the omentum all around. Its main connection with the patient appeared to be the infundibulo-pelvic ligament. Its connection with the uterus was so friable that, upon slight traction being made, it tore loose and left a mass of apparently necrotic, bleeding omentum in my hand, extending behind the uterus to the opposite side. In removing this unhealthy tissue, I had to leave a short stretch of intestine without any omentum. The interior of the patient's pelvis seemed pretty well torn to pieces, and filled with ligatures. The abdominal cavity had been open about two hours, and considerable blood had been lost.

She has, however, made a good recovery, except that over-exertion or attacks of bowel complaint bring on a pain in the iliac region. The uterus is in a normal position, and hence cured of its prolapse, but not yet movable in the pelvis.

Professor L. L. McArthur: I have had the pleasure of examining this specimen, and find it to consist of a rather rare growth for this region, that is, a papilloma. By a papilloma, one usually understands a structure consisting of connective tissue and capillary blood-vessels, covered with epithelium, that may be either simple or compound, according to location; in fact, resembling the structure of the papillæ of the skin. Papillomata are found very commonly about the integument. It was formerly thought that papillomata were only to be found where papillæ were to be found, as in the skin, the mucous membrane of the mouth, etc. But that view was soon changed by finding that the papillomata may develop from any epithelial surface. They are exceedingly rare when found on a serous surface, and but few are on record as being found in the position in which this specimen was found. In the "Encyclopædia of Obstetrics and Gynæcology" a few cases have been collected by Olshausen, five I believe, the first of which was presented by Prochaska. Papillomata of the peritoneum or of the abdominal cavity occur most frequently at that portion of the ovary that is not covered by peritoneum, or only partially covered by peritoneum. They vary in shape, consisting sometimes of a single finger-like projection, at other times presenting many ramifications. In this case the tumor, when presented to me, resembled very much the appearance of a little branch of cauliflower. I do not think it could be likened to anything that it resembled more closely. The tumor measured about three centimetres in length, and two and a half centimetres in thickness. I made microscopic sections of it and present one to-night. An instructive as well as interesting peculiarity about this is the fact that it is infiltrated with tubercular foci; there are miliary tubercles scattered throughout the papilloma. Very few cases have been recorded in which neoplasms are also infected with acute or chronic infectious diseases, hence it is worth placing on record.

Lange has recently called attention to the fact that new growths may take place upon diseased surfaces, and also calls attention to the fact that carcinoma has developed from a surface that in the first place was purely of a syphilitic character. And here we have a simple growth showing infiltrations that are decidely malignant. In reporting the four cases he has collected, Olshausen makes an interesting comment upon the fact that papillomata most frequently develop in the uncovered surface of the ovary *because* they are most likely to be exposed at that point to the action of irritant matter that may escape at the fimbriated extremity of the Fallopian tube, as in gonorrhœal infection, in the case of tubercular pus, or of those other irritating materials that may gain admission, and explains their growing in this situation in this way. We know that papillomata do grow where the irritation is marked and continued, as for instance in the prepuce, when gonorrhœa is present, or at the vulva.

I present this as being instructive and interesting.

The PRESIDENT also reported "Two Cases of Alveolar Sarcoma of the Uterus," and said: I have had the fortune during the past summer to meet three cases of soft, or alveolar sarcoma of the uterus. I believe there are only about 100 cases on record. I have two here; the third uterus went to pieces on extraction. This makes four cases of this kind I have seen. Both of these uteri were taken from virgins, forty-eight years old. They both presented to vaginal indagation almost the same condition, except that in one the disease extended to the external os, and both presented the same symptoms, namely, hæmorrhage and pain. In neither case was the discharge very offensive. Both took from one-half to one grain of morphine a day. Both had been curetted a week before the operation, in order to reduce the uterus and render their removal *per vaginam* practicable. One of the patients, Miss M——n, had been curetted three times before. She had hæmorrhage after hæmorrhage, each one threatening to kill her, and when she came to me she looked like a person dying with consumption, I removed the uterus with great difficulty, as the vagina was contracted from the use of styptics. The stumps healed nicely, and the patient seemed well. One morning she refused to take her food and medicine, and died the same evening. We had been unable by threats or persuasion to get her to take anything but a little rice or milk and lime-water. I suppose there is no doubt that, if she had been operated upon earlier, her life would have been saved. Dr. Wing made a postmortem examination. He found the stumps were in good condition, and declared the cause of death to be inanition. The most important predisposing cause of death was probably the exhausting hæmorrhages previous to the operation.

The other patient, Mrs. Sh——, was stronger, and got well without a bad symptom.

SARCOMA OF THE OVARY.

This specimen I bring because it is fresh. It was taken out yesterday, and is a beautiful specimen of sarcoma of the ovary. There were no adhesions nor pain. It was first discovered in July, since when it has grown so rapidly as to be the size of a man's head.

Professor JAGGARD said that sarcoma of the uterus and ovaries was a comparatively rare morbid state. While he did not wish to cast aspersions upon the adequacy of the President's diagnostic methods, still he thought the well-established custom of this and similar societies ought to be strictly adhered to in the matter. Either sections of the neoplasm, showing plainly its alleged character, ought to be exhibited, or a report by a qualified pathological anatomist should be read. The evidence upon which the diagnosis of sarcoma in the specimens presented rests, in so far as it is contained in the President's remarks, is insufficient. This material, in the absence of such evidence, is well-nigh valueless for the purposes of comparative study and record. Dr. Jaggard was moved to utter these ungracious words by the fact that an enormous amount of really valuable material had been wasted, not only at this but also at former meetings of the Society.

Dr. J. FRANK (present by invitation) presented a vesical calculus formed around a silk-worm gut suture, inserted into the vesico-vaginal septum.

These pieces of calculus were removed from a woman about four weeks ago. She was operated upon seven months ago, at the County Hospital, for vesico-uterine fistula. The stitches were taken with silk-worm gut, and left in. The woman made a nice recovery, and the bladder held its contents for three months, when the urine commenced dribbling again through the vagina. The woman became very much distressed, and upon examination I found that from the site of the stitches a calculus had formed within the bladder, about half the size of a billiard ball. I removed the calculus, and in one of the pieces I have here there is one of the stitches. The woman made a good recovery.

Dr. HENRY P. NEWMAN read a paper entitled "Alexander's Operation, with Report of Cases."

My object in presenting a necessarily brief report of seven recent cases of Alexander's operation is, first, to invite the consideration of this Society to the operation of shortening the round ligaments for uterine displacements.

Second, to refer briefly to the manner of operating, suggesting some modificatious in the *technique*, which have been applied in five consecutive cases.

The operation, as is well known, was given to the profession early in 1882, more than six years ago, by Wm. Alexander, of Liverpool.

Since then it has been performed upward of three hundred times, as reported by different operators, with very satisfactory results. Notwithstanding these promising reports, and all that has been said and written by those who are familiar with the operation and convinced of its utility, there has been much adverse criticism from high authorities in gynæcological literature.

The clinical evidence thus far obtained is decidedly in support of the utility of the operation.

Dr. Alexander himself, in his brilliant report of eighty cases in hospital and private practice, has demonstrated the fact that the anatomical cure of retroflexions and retroversions by shortening the round ligaments is a most certain result of the operation; and in cases of prolapsus, when he operated upon the perineum at the same time, he was equally successful with what he termed the "double operation."

The unpublished cases of our president, Dr. Byford, and those reported by Dr. J. H. Kellogg, of Michigan, are confirmatory of the success of the procedure in the hands of American operators.

Due credit should be given to Dr. Doléris, Paris, France, for his excellent paper on "Combined Operations for the Relief of Uterine Deviations or Displacements," wherein he classifies the factors which unite in maintaining the pelvic equilibrium, and endeavors to point out the indications for the performance of the Alexander and other accessory operations.

In my own cases, I had early been convinced of the advisability of using all auxiliary therapeutic measures, preparatory to shortening the round ligaments, as will be seen in the appended cases.

In three of the seven, the cervix and perineum were repaired; in one, cervix alone; in one, anterior and posterior colporrhaphy were done, and in one, scraping or curetting the uterus for vegetations, and in each instance shortening of the round ligaments was not resorted to until other feasible methods had been given a fair trial.

This manner of procedure seems to me especially demanded in cases of procidentia, particularly when there is a capacious vagina, a relaxed anterior or posterior vaginal wall, or torn perineum; in other words, where anterior and posterior colporrhaphy and perineorrhaphy are indicated. While I would not say that, in these complicated cases, no benefit could result from Alexander's operation alone, I believe it to be good surgery to fortify it by such auxiliary measures as are known to remedy the tissues at fault, and not to over-estimate the proper function of the round ligaments.

For similar reasons I should favor the treatment of long-standing or aggravated cases of flexions accompanying backward or forward misplacements, by such methods as that devised by Professor A. Reeves Jackson and others, for restoring the integrity of the uterine walls.

The seven cases which I shall briefly outline, are of such recent date that I publish them, in this connection, merely to illustrate a modified method of operating, which has been, in the main, suggested by Dr. J. Frank, attending surgeon to the St. Elizabeth's Hospital, and its adaptability verified by dissections upon the cadaver.

I begin the operation by cutting directly down upon the internal inguinal ring, using the superficial epigastric vein, and beneath this the illio-inguinal nerve, as guides, lying as they do directly over the canal of Nuck.

The superficial landmarks are the same as are usually followed in this operation, *i. e.*, Poupart's ligament, spine of the pubis, and the anterior superior spinous process of the ilium.

The superficial epigastric vein, as well as the internal ring, is situated midway between the two last-named bony prominences.

The primary incision, beginning just above the spine of the pubis, and extending one and a half inches or more upward and outward, parallel with Poupart's ligament, comes at once upon the vein, which will be found in the subcutaneous fat, near the upper third of the cut.

This vein, though often the size of a goose-quill, having no material importance except as a guide, is ligated and cut across leaving the ligature long, for facility in locating.

On reaching the underlying external oblique aponeurosis, which is easily recognized by its glistening white appearance, it is cut through in the course of its longitudinal fibres, and corresponding with the superficial incision.

The ilio-inguinal nerve is now seen lying along the course of the canal, just beneath or mingling with the lower marginal fibres of the transversalis muscles.

It is just beneath the point where the epigastric vein crosses the course of the ilio-inguinal nerve that the round ligament can be readily defined.

If the muscular and fibrous tissues surrounding the cord are picked up *en masse* by forceps, and slightly pulled upward, and then spread over the thumb or finger inserted beneath, the whitish, slightly flattened, cord-like ligament is seen.

It is now isolated, largely by the fingers, and made to run, care being used not to injure the ilio-inguinal nerve, which should be drawn to one side.

The ligaments should now be pulled upon to their full extent, or until the uterus is drawn upward into an erect or somewhat anteverted position, as is determined by an assistant's finger in the vagina, who either holds the uterus in this position by a sound *in utero* or immediately inserts a preivously fitted pessary.

The ligament is stitched to each side of the canal by a suture of silk-worm or chromic acid catgut, passing the stitch through one pillar of the canal, then into the taut ligament and along its longitudinal fibres, and returning it through the pillar of the same side.

The *canal* is then closed by two or three sutures of the same material, made to include a small portion of the cord, but drawn only sufficiently tight to approximate the edges.

The loop of the ligament, instead of being excised, is secured by a firm stitch uniting the proximate ends, and then folded into the wound after the method of Dr. Burt, of Boston.

The wound is now closed, the customary drainage-tube inserted—supplemented by prepared iodoform wicking drawn into the depth of the tube for capillary drainage.

The ligament of the opposite side—which, after being pulled out and held by a sponge or pledget of gauze inserted beneath, was covered with antiseptic gauze and left—is now treated in the same manner.

Strict antiseptic precautions having been used in all details of the operation, permanent iodoform dressings are applied, and the wound exposed only in case of rise in temperature or excessive soiling of the dressings, until the fourth or fifth day, when the drainage-tube is removed and the wicking substituted.

Patient is kept in bed about four weeks, great caution insisted upon in getting up, and for four to five months following no severe exertion is allowed.

The pessary is retained during this period, and an elastic abdominal support worn.

CASE I.—Mrs. L., age 33, married twelve years; one child, ten years of age. No miscarriages. Puberty at fifteen.

Slight laceration of cervix and perineum. Uterus large, prolapsed, and retroverted; depth four and one-half inches. Troubled with menorrhagia and metrorrhagia for several months, the introduction of the sound invariably causing hæmorrhage.

Suffered much pain at the menstrual period, and was never entirely free from distress in the pelvic organs.

In March last, I curetted the uterus for vegetations, removing a large quantity.

April 21, I performed Alexander's operation. The wound did well, and patient was able to sit up at the end of the fourth week.

In the ninth week, when she returned to her home in Nebraska, the uterus was found in good position, well up in the pelvic cavity. When last heard from, August 1, she reported herself in better health than for years, and doing her own housework, which she had not been able to do for eight years.

CASE II.—Mrs. W., 35 years of age. Had borne eight children, and had two miscarriages. Had been under medical treatment constantly for two years, and had not been well for ten.

Uterus retroverted and strongly retroflexed; laceration of cervix and perineum.

February 6, 1888, was operated upon by Dr. R. N. Hall, for lacerations of cervix and perineum, and the uterus was dilated at the same time for the purpose of straightening.

The flexion returning, however, I was asked to do Alexander's operation.

May 31, the round ligaments were shortened. Some difficulty was experienced in picking up the ligaments, necessitating tearing of the tissues.

There was some sloughing in this case, referred partly to the tearing of the tissues in operating, and in part to the patient herself, who persisted in continually disturbing the dressings.

She was an extremely unmanageable patient, and on June 19 left the hospital without the knowledge of her attending physician, who abandoned the case, and consequently nothing further has been learned of her condition, except what may be inferred from the fact that the doctor in passing has seen her around the house.

CASE III.—Mrs. P., age 36 years, had suffered for eleven years from prolapse or procidentia of uterus; ovaries large, tender, and prolapsed, so that pessary was tolerated with great difficulty.

Was able to do little or nothing in the way of household duties, though the mother of a large family.

Menses irregular, profuse, and painful. When first seen, early in May last, the uterus was enlarged and heavy, appearing at the vulva, and the effort of straining or bearing down forced it without the vaginal orifice.

Vagina was capacious, and rectal and vesical walls greatly relaxed.

The operations of anterior and posterior colporrhaphy were advised, and a few weeks after performed, with only partial relief.

The round ligaments were shortened August 16, 1888. Wound healed promptly by first intention.

In fourth week patient was up and about, and left the hospital at the end of the fifth, feeling quite well, with the uterus retained in normal position.

She was seen six weeks after the operation at her home, and expressed herself as still feeling well. Had little or no pain at last menstrual period, and was engaged in light household duties.

Examination showed uterus held well up, and scarcely resting upon the Hodge pessary which she had been instructed to wear.

CASE IV.—Mrs. E., age 23, married four years. One child, two miscarriages. Had suffered three years with prolapsus and subinvolution following the birth of her child. She also had lacerated cervix and perineum, and suffered more or less constant dragging pain at the menses and during the month. Flow profuse, irregular, and followed by leucorrhœa; reflex dyspeptic symptoms of great annoyance, not relieved by the usual remedies.

June 1, I operated upon the cervix and perineum, with only slight relief of the reflex symptoms.

August 24, I shortened the round ligaments nearly four inches.

The operation was followed by no unpleasant symptoms, and at the end of the third week the patient was allowed to sit up, and returned to her home at the end of the fourth week.

Five weeks after the operation has none of her former distress in back and sides; dyspeptic symptoms are rapidly improving.

The uterus remains in excellent position, and involution is taking place rapidly.

CASE V.—Mrs. N., age 29 years, eleven years married. Three children, two miscarriages. Nine years ago began to have backache and bearing-down pains. From year to year these have become worse, until she has been almost incapacitated for her household duties. When first examined, some eight months ago, the uterus was found heavy, prolapsed, and retroverted; cervix and perineum badly torn; both ovaries enlarged, prolapsed and tender, so that no pessary could be endured.

In June last, the double operation upon cervix and perineum was performed; and

Alexander's operation on August 25, at her home. Though lacking in conveniences and trained attendants, patient's recovery was rapid and satisfactory, requiring but little more care and attention than an ordinary cervical or perineal operation.

In the fifth week after the operation I find the woman about the house and attending to her household duties, but exercising caution as she has been strictly enjoined.

Instead of the prolapsed and retroverted uterus, and ovaries prolapsed, tender, and enlarged, we now find both uterus and ovaries drawn well up, the latter beyond the reach of the finger.

No pain is experienced, and the patient feels herself recovered, though showing some anæmia and weakness from the confinement incident to the two eperations and the result of her former condition.

CASE VI.—Mrs. G., age 34, married three years; no children. Former occupation, laundress and seamstress. Has suffered with retroversion and prolapsus for fifteen years, with distressing pains in back, with dysmenorrhœa, and irregular menses followed by leucorrhœa.

At her own urgent request, Alexander's operation was done the 27th of August last. In this case the healing was so prompt that, being obliged to leave the city for a short time, I yielded to the temptation to remove the stitches—in this instance, silk—on the fifth day. I left the case in care of Dr. C. W. Leigh, who reported satisfactory progress until subsequent dressing on seventh day.

On this day, some sudden movement in bed resulted in slight gaping of the wound upon left side.

On account of this, the patient has been kept in bed for the wound to heal by granulation. I expect no trouble from the accident. The uterus is held in good position, and wound in the right side is healed firmly. In other respects, she is in excellent condition and feels well .

CASE VII.—Mrs. S., age 27, married five years; three children. Had retroversion of uterus and ovarian prolapse. Menses always painful, and often prolonged eight days. Pain in back. Uterus subinvoluted, cervix and perineum torn. Leucorrhœa profuse and constant. Patient very much reduced and unable to work.

Trachelorrhaphy and perineorrhaphy were performed in June, and a uterine support subsequently used, without relieving her distressing symptoms.

The round ligaments were shortened four inches on September 11.

Union by first intention ; uterus held well in position and everthing promises speedy recovery with good results.

The last five cases are reported simply to illustrate the anatomical results of the modified method of operating.

In each instance the healing has been by first intention, the amount of surgical disturbance nil, and the results, to date, all that could be desired.

The advantages claimed are the readiness with which the round ligaments are found and separated from their environment, necessitating no tearing of the surrounding parts and disturbance of the tissues about the external inguinal ring, and the terminal filaments of the broad ligament at its pubic end, with the least possible danger of hernia as an after result.

Should it be possible, as has been suggested by Dr. Frank, and, as I learn from late readings, is practiced by Dr. Doléris, to do without the drainage-tube, the operation and after-care would be even more simplified and devoid of some of its now possible accidents.

Professor W. W. JAGGARD: The construction of the indications for Alexander's operation is a curious study. An eminent gynæcologist, especially prominent as a skillful operator, resident in a metropolis, with a large and uncommonly exacting clientéle, writes: "For unmanageable cases of posterior displacement—which I have not yet met with—a new operation has been devised, that of shortening the round ligaments. But since this operation is in its infancy, and I thus far have not had any need of resorting to it, I cannot yet recommend it to you" (Goodell, "Lessons in Gynæcology," 1887, p. 165). On the other hand, a practitioner at Land's End sees the need of this operation one hundred times in three years! This difference in opinion and practice requires no comment, and the moral is perfectly obvious. It reminds one of Billroth's remarks on the unjustifiable frequency of excision of the pylorus for cancer. After he had determined for himself the feasibility of the operation, he waited just four years before he saw a case that indicated the procedure. Within six months of the publication of this case, the operation had been performed some half-score of times by minor surgeons.

In the cases reported this evening, two conditions were uniformly present—in each case the uterus was perfectly movable and easily replaceable.

Before resorting to such an heroic measure as Alexander's operation, it seems to me to have been the plain duty of the operator to attempt persistently the bimanual correction of the displacement, and the retention of the uterus in normal position by a suitable pessary. It is not sufficient to say that "pessary treatment" had been unsuccessfully tried before the cases came under his observation. Nowadays, it is the surgeon's duty, not merely to perform an operation, but also to determine for himself the nature and force of the indication.

For myself, I have as yet encountered no case presenting a clear indication for shortening of the round ligaments, and, hence, I have never performed the operation. But I have frequently seen the operation performed by others. In one case, the round ligaments were shortened to correct the retroversion of an infantile uterus, no bigger than the end of your thumb, in a virgin of eighteen. In two cases the retroversion was due to subinvolution, and the operation was done within twelve months of the antecedent confinement.

I desire to limit my remarks to the single point of indication for the operation. I have no intention, at present, to discuss the absolute merits of the procedure, nor its claims in comparison with the devices of Emmet, Schücking, and others. In the interesting paper read this evening, in my opinion, the indication for Alexander's operation is adequately sustained in not a single case.

Dr. J. FRANK: There is a question in my mind as to whether the operation is performed for disease of the uterus or of the round ligaments. It can be either, although the trouble has always been referred to the uterus and not to the round ligaments. The uterus can be retroverted because the round ligaments are diseased, or the uterus may be enlarged and drag upon the round ligaments, and still the fibres may be in a healthy condition. The round ligaments are made up mostly of elastic fibres, and if there is degeneration or atrophy, I do not think the operation would be useful. I think, just as Dr. Jaggard does, that much can be accomplished with pessaries.

There is another question about this operation. Will the cicatrix that is made by the incision last? Won't it be the same as in hernia operations; after awhile the cicatrix will begin to atrophy and soften, and allow the uterus to go back into its place.

The PRESIDENT: I have, so far, operated in twenty-one cases, and believe in the operation as at first, but do not operate as much. I have had two failures. They were failures because I ought not to have performed the operation. In one case there was salpingitis with adhesions; in the other case there was an enlarged ovary and hydro-salpinx. The case of salpingitis resulted in slight peritonitis, with a temperature of 102 Fahr. for several days. I took out the pessary at the end of six weeks, and at the end of two months, when she was at her housework, retroversion and peritonitis recurred. The uterus can now be easily held up by a pessary, while it could not before the operation, and will remain up after the pessary is removed until some strain forces it down. At present it is in position without a pessary. I think the uterus would stay in good position if the patient would allow me to remove the enlarged tube, but as it is, she is better off than before the operation. In all of my operations, I now leave the pessary in place from six months to a year. I had another case which might be called a failure, as an inguinal hernia resulted. The patient had been to some of the most eminent gynæcologists in Chicago, one of whom wanted to remove the ovaries. She could not tolerate a pessary of any description. Now she wears a pessary without trouble; the uterus is up, and she is one of the most grateful patients I have, in spite of her rupture. On account of this hernia, I threw aside my catgut, and I now use none but silk-worm gut sutures, and leave those that close the canal and hold the ligament.

One great drawback to the operation is that the patients are not always immediately cured of symptoms. If there have been pelvic contractions or adhesions, there may be a dragging on these for some months. The first case I had was of this kind. The pessary was left in two months. She complained long afterward of a dragging on the groins, but at the end of a year she could, for the first time in several years, do her own housework, and take care of her home. She had previously had gynæcological treatment for five years, the last two by myself.

In regard to the technique of the operation, I think that Dr. Jaggard is right in saying that many bungling operations are done, but that has nothing to do with the indications for so new an operation. It is possible to do a good operation. Suppuration should be exceptional. I use drainage for twenty-four hours, merely to let out the bloody oozing.

It is objected that the round ligament is not a normal support of the uterus. We do not shorten the round ligaments to support the uterus in cases of retroversion, as is so generally misunderstood; we merely turn the fundus forward and depend upon the normal abdominal pressure to hold it forward.

Of course, the operation is only for the few cases in which the displacement causes symptoms, and in which it can be replaced, but cannot be permanently retained in position by less available or less dangerous means.

---

ANNUAL MEETING, FRIDAY, OCTOBER 19, 1888.

The President, HENRY T. BYFORD, M. D., in the chair.

After the reports of the secretary, treasurer, and editor were read and accepted, the retiring president read a paper entitled "Twelve Months of Abdominal and Vaginal Section."

The following officers for the year 1888–1889 were then elected:

President, Charles T. Parkes.
First Vice-President, E. J. Doering.
Second Vice-President, E. C. Dudley.
Secretary, Edward Warren Sawyer.
Treasurer, F. E. Waxham.
Editor, W. W. Jaggard.

The Society then adjourned.

